# Impact of valproate co-medication and age on lurasidone exposure: a population pharmacokinetic study and real-world evaluation in Chinese psychiatric inpatients

**DOI:** 10.3389/fphar.2026.1810528

**Published:** 2026-05-12

**Authors:** Ye Yang, Haoyang Lu, Tao Xiao, Xiaojia Ni, Zhanzhang Wang, Yuqing Chen, Lijing Dai, Emei Song, Fanghua Su, Yuguan Wen

**Affiliations:** 1 Department of Pharmacy, Guangdong Sanjiu Brain Hospital, Guangzhou, Guangdong, China; 2 Department of Pharmacy, The Affiliated Brain Hospital of Guangzhou Medical University, Guangzhou, Guangdong, China; 3 Key Laboratory of Neurogenetics and Channelopathies of Guangdong Province and the Ministry of Education of China, Guangzhou Medical University, Guangzhou, Guangdong, China; 4 Department of Clinical Research, The Affiliated Guangdong Second Provincial General Hospital of Jinan University, Guangzhou, Guangdong, China; 5 Department of Pharmacy, Guangdong North Third People’s Hospital, Shaoguan, Guangdong, China

**Keywords:** Chinese psychiatric inpatients, lurasidone, NONMEM, population pharmacokinetics, therapeutic drug monitoring

## Abstract

**Objectives:**

To characterize the population pharmacokinetics (PPK) of lurasidone in Chinese psychiatric inpatients and to quantify the sources of inter-individual variability, with a specific focus on the impact of valproate (VPA) co-medication and age on drug exposure.

**Methods:**

Routine therapeutic drug monitoring (TDM) data were collected from 156 patients (providing 212 serum concentrations). A PPK model was developed utilizing a nonlinear mixed-effects modeling approach (NONMEM). Model performance was assessed via goodness-of-fit plots, normalized prediction distribution errors (NPDE), and bootstrap analysis. Additionally, model-based simulations were performed to estimate concentration profiles stratified by age and VPA co-medication.

**Results:**

A one-compartment model with first-order absorption and elimination best described the data. The typical estimate for apparent clearance (CL/F) was 339 L·h^-1^. Age and concomitant VPA were identified as significant covariates. VPA co-administration increased CL/F by 47.7%, leading to significantly reduced systemic exposure. Notably, the majority of observed steady-state concentrations (3–11 ng/mL) fell below the lower limit of the AGNP reference range.

**Conclusion:**

The pharmacokinetics of lurasidone in Chinese inpatients were effectively characterized by the established PPK model. Lurasidone exposure in this population is generally lower than the AGNP reference range, particularly in patients receiving VPA. Age and VPA co-medication are critical determinants of CL/F. These findings suggest that TDM interpretation should be adjusted for these factors, and optimal concentration targets for Chinese patients may need to be revisited.

## 1 Introduction

Lurasidone is a second-generation antipsychotic with a unique receptor-binding profile, functioning as an antagonist at dopamine D_2_, serotonin 5-HT_2A_, and 5-HT_7_ receptors, and as a partial agonist at 5-HT_1A_ receptors (Gu et al., 2023). Approved by the U.S. FDA in 2010 and introduced in China in 2019, lurasidone is primarily indicated for the treatment of schizophrenia and bipolar disorder (Miura et al., 2023).

Within the therapeutic dose range (20–160 mg/day), lurasidone exhibits linear pharmacokinetics and reaches steady state after approximately 7 days. It is highly protein-bound (∼99%) and primarily metabolized by CYP3A4 (Hu et al., 2017; Greenberg and Citrome, 2017). Food significantly enhances absorption, increasing systemic exposure by approximately 2- to 3-fold under fed conditions (Preskorn et al., 2013). The active metabolite, ID-14283, attains peak concentrations approximately 25% of those of the parent drug, suggesting the parent compound contributes the predominant pharmacological activity. According to the Arbeitsgemeinschaft für Neuropsychopharmakologie und Pharmakopsychiatrie (AGNP) guidelines, the recommended therapeutic reference range is 15–40 ng/mL (Hiemke et al., 2018). In our previous therapeutic drug monitoring (TDM) study, we observed that lurasidone serum concentrations in Chinese psychiatric patients were frequently below the recommended AGNP lower threshold (15 ng/mL) despite receiving standard doses. Notably, preliminary analyses identified age and concomitant valproate (VPA) as significant factors influencing the concentration-to-dose (C/D) ratio (Yang et al., 2024). However, the mechanism and clinical magnitude of this interaction remain to be fully elucidated. Polypharmacy is the rule rather than the exception in psychiatric practice, and VPA is a cornerstone mood stabilizer frequently co-prescribed with atypical antipsychotics. Recent clinical evidence has confirmed that adding lurasidone to ongoing therapy with mood stabilizers like VPA or lithium is both effective and well-tolerated (Tocco and Mao, 2024). While VPA is classically characterized as a broad-spectrum enzyme inhibitor, its specific impact on lurasidone, a CYP3A4 substrate, is ambiguous, with conflicting hypotheses pointing towards potential PXR-mediated enzyme induction or protein binding displacement (Cerveny et al., 2007; Diaz et al., 2014). Given the high inter-individual variability and the risk of exposure below the lower limit of the therapeutic reference range (15 ng/mL), a rigorous population pharmacokinetic (PPK) analysis is required to quantify these effects and optimize precision dosing strategies for the Chinese population.

Although the AGNP guidelines recommend a therapeutic reference range of 15–40 ng/mL, emerging evidence suggests significant inter-ethnic variability in lurasidone pharmacokinetics (Zhang, 2022). Preliminary observations in Chinese clinical practice indicate that patients often exhibit lower serum concentrations despite receiving standard doses (Yang et al., 2024; Dai et al., 2026; Huang and Lin, 2022). Furthermore, the potential impact of common co-medications, particularly VPA, remains under-investigated in this population. Validating these factors is crucial for optimizing precision medicine (Yang et al., 2024). This study aimed to develop a PPK model to quantify the influence of age and VPA on lurasidone disposition and to critically evaluate the attainability of current therapeutic targets under real-world conditions.

## 2 Methods

### 2.1 Study design and ethics

This study was a retrospective PPK analysis utilizing routine TDM data from Chinese psychiatric inpatients treated with lurasidone. Serum samples were collected as part of routine clinical care and analyzed in the hospital’s central laboratory using validated assays. The study protocol was reviewed and approved by the Institutional Ethics Committee of The Affiliated Brain Hospital of Guangzhou Medical University (Approval No. 2021027). Given that all data were anonymized and extracted from retrospective medical records, informed consent was waived in accordance with national regulations and the 2013 revision of the Declaration of Helsinki.

### 2.2 Patients and data collection

Demographic and clinical data for all lurasidone-treated inpatients were retrospectively retrieved from the hospital’s Electronic Medical Record (EMR) and Laboratory Information System (LIS). The collected variables encompassed demographic characteristics (sex, age, height, weight), dosing history (dosage regimens, dosing interval, time of last dose, and concomitant medications), and lurasidone serum concentrations obtained via routine TDM. Additionally, biochemical markers of hepatic and renal function were recorded, including alanine aminotransferase (ALT), aspartate aminotransferase (AST), total bilirubin (TBIL), albumin (ALB), urea, serum creatinine (Scr) and creatinine clearance (CrCL), CrCL was estimated using the Cockcroft-Gault formula.

### 2.3 Inclusion and exclusion criteria

Eligible subjects were hospitalized Chinese patients (of Han ethnicity) who received oral lurasidone, had documented TDM records with dosing regimens, and a psychiatric diagnosis according to International Classification of Diseases, 10th Revision (ICD-10) criteria. Exclusion criteria included poor medication adherence, incomplete demographic or dosing records, pregnancy, serum concentrations of zero or outside the assay’s quantification range, severe hepatic impairment (Child-Pugh score≥10), severe renal impairment (creatinine clearance≤30 mL/min), or unstable clinical conditions. All data were anonymized before analysis.

### 2.4 Determination of lurasidone concentrations

Serum lurasidone concentrations were measured using a validated high-performance liquid chromatography-tandem mass spectrometry (HPLC-MS/MS) method (Yang and Ni, 2023). The HPLC-MS/MS method was fully validated according to the Chinese Pharmacopoeia guidelines. The calibration curve was linear over 1–200 ng/mL (*r* ^2^ = 0.9997). The lower limit of quantification (LLOQ) was established at 1.0 ng/mL (intra- and inter-batch precision CV <10%, accuracy RE within ±3%). The internal standard-normalized matrix factors showed no significant matrix effect (CV <12%), even when evaluated in 3% hemolyzed and 6% hyperlipidemic sera. Comprehensive stability tests using low and high QC samples confirmed that lurasidone in human serum was stable at room temperature for 24 h, across 5 freeze-thaw cycles, and under long-term storage at −20 °C for 41 days (all RE within ±6%).

### 2.5 PPK modeling and covariate analysis

PPK modeling was performed using NONMEM (version 7.3; ICON Development Solutions) with the first-order conditional estimation with interaction (FOCE-I) method (Keizer et al., 2013; Mould et al., 2013). Model development proceeded sequentially by defining the structural, statistical, and covariate submodels. Due to the sparse sampling design (predominantly trough levels), the absorption rate constant (K_a_) could not be reliably estimated and was fixed at 0.679 h^-1^ based on the reported literature (Hu et al., 2017). This practice of fixing parameters in sparse data environments is supported by established pharmacometric guidelines (Savic and Karlsson, 2009; Ette and Williams, 2004). Additionally, a sensitivity analysis was conducted to assess the impact of fixing the K_a_ by evaluating a ±20% variation from the fixed value (Supplementary Table S1). This approach assumes constant absorption kinetics across the population, attributing inter-individual variability primarily to apparent clearance (CL/F) and apparent volume of distribution (V/F). Interindividual variability (IIV) in CL/F and V/F was modeled using exponential error models. Residual unexplained variability (RUV) was evaluated using exponential error models, with the optimal model selected based on the lowest objective function value (OFV) and inspection of conditional weighted residuals (CWRES) plots.

Potential covariates, selected for biological plausibility and clinical relevance, included demographics, hepatic and renal biomarkers, and concomitant VPA use. Covariate screening followed a stepwise approach: forward inclusion (ΔOFV >3.84, p < 0.05) and backward elimination (ΔOFV >6.63, p < 0.01) to retain significant covariates in the final model (Jonsson and Karlsson, 1998).

Continuous covariates were parameterized using a power model centered on the population median: $P_{i}=P_{\text{pop}}\times {\left(\frac{{\text{COV}}_{i}}{{\text{COV}}_{\text{median}}}\right)}^{\theta_{\text{cov}}}$ , where $P_{i}$ is the individual parameter value, $P_{\text{pop}}$ is the typical population estimate, ${\text{COV}}_{i}$ is the covariate value for subject i , and $\theta_{\text{cov}}$ is the estimated exponent describing the covariate effect. Categorical covariates were implemented as: $P_{i}=P_{\text{pop}}\times \left(1+\theta_{\text{cov}}\times {\text{COV}}_{i}\right)$ , where ${\text{COV}}_{i}$ = 0 for the reference category and 1 for the comparison group. Additionally, a Spearman correlation analysis between concurrent VPA serum concentrations and the final model ETAs on CL/F was conducted in a subset of 39 paired observations to justify the binary covariate approach (Supplementary Figure S1A).

### 2.6 Model evaluation

Model adequacy was evaluated through graphical diagnostics, simulation-based methods, and resampling techniques (Nguyen et al., 2017). Goodness-of-fit (GOF) plots included observed concentrations (DV) versus population predictions (PRED), DV versus individual predictions (IPRED), conditional weighted residuals (CWRES) versus PRED, and CWRES versus time after last dose. Normalized prediction distribution errors (NPDE) were employed to assess bias and variance across time and concentration ranges (Comets et al., 2008). Model robustness and parameter precision were evaluated using non-parametric bootstrap resampling (n = 1000), with bootstrap medians and 95% confidence intervals compared to final parameter estimates to confirm stability. In addition to the median and 95% confidence intervals (CIs), the relative standard error (RSE%) derived from the bootstrap results was calculated to further assess the precision of the parameter estimates.

### 2.7 Model-based simulations

Model-based simulations were conducted using the final PPK model in NONMEM to generate virtual cohorts stratified by age group (adolescent [13–17 years], adult [18–64 years], elderly [≥65 years]) and VPA co-administration (monotherapy vs. concomitant VPA). Steady-state trough concentrations were simulated for once-daily oral doses of 20, 40, 60, 80, and 120 mg. Probability of target attainment (PTA) was calculated for the AGNP therapeutic window (15–40 ng/mL) and an exploratory internal reference range (3–11 ng/mL), and summarized by dose, age group, and VPA status.

## 3 Results

### 3.1 General patient characteristics

A total of 156 patients contributing 212 lurasidone serum samples were included in the analysis. The median (range) lurasidone concentration was 6.3 (1.0–44.5) ng·mL^-1^, with a mean ± SD of 8.57 ± 7.81 ng/mL. The median age was 22 years (range 13–70), and 69% of the cohort were female. Demographically, the study population consisted of 60 adolescents (13–17 years), 92 adults (18–64 years), and 4 elderly patients (≥65 years), as detailed in Supplementary Table S2. The median (range) administered dose of lurasidone for the overall cohort was 60 (20–120) mg/day. Across the age subgroups, the median doses were 60 (20–80) mg/day for adolescents, 60 (20–120) mg/day for adults, and 50 (20–80) mg/day for the elderly, as detailed in Supplementary Table S3. The mean BMI was 25.2 ± 6.4 kg/m^2^. Concomitant VPA and lithium carbonate were prescribed to 28% and 44% of patients, respectively. Hepatic and renal function parameters were generally within reference ranges (Table 1).

**TABLE 1:** General information on lurasidone PPK included patients.

Information and materials	Case number (%)	Median (range)	Mean ± SD
Patient	156	—	—
concentration (ng/mL)	212	6.30 (1.00–44.53)	8.57 ± 7.81
Gender (male/Female)	47/109	—	—
Age (year)	—	22.00 (13.00–70.00)	25.76 ± 12.86
Height (cm)	—	164.00 (148.00–190.00)	165.18 ± 8.78
Weight (kg)	—	65.50 (36.00–138.00)	69.72 ± 22.28
BMI(kg/m^2^)	—	23.45 (15.40–43.40)	25.24 ± 6.44
Concomitant VPA, n (%)	59 (28%)	—	—
Concomitant lithium carbonate, n (%)	93 (44%)	—	—
ALT (U·L^-1^)	—	15.00 (4.00–219.00)	23.89 ± 24.28
AST (U·L^-1^)	—	18.00 (6.00–143.00)	20.50 ± 13.14
TBIL (μmol·L^-1^)	—	10.30 (2.80–35.00)	11.35 ± 5.23
ALB (g·L^-1^)	—	38.00 (3.80–56.00)	38.73 ± 4.76
Urea (mmol·L^-1^)	—	3.59 (1.36–8.34)	3.87 ± 1.14
Scr(umol·L^-1^)	—	57.00 (34.00–115.00)	60.42 ± 14.94
CrCL (mL/min)	​	145.06 (41.85–418.53)	150.57 ± 57.37

### 3.2 Base model development

A one-compartment model with first-order absorption and linear elimination was selected to describe the pharmacokinetics of lurasidone. Given that the current dataset consisted primarily of sparse TDM samples collected at steady-state troughs, absorption-related parameters could not be uniquely identified solely from the data. Consequently, the absorption rate constant $K_{a}$ was fixed at 0.679 1/h. This value was selected to align the model-predicted absorption phase with the reported time to maximum concentration ( $T_{\max}$ ∼1.0–3.0 h) in the Chinese population (Hu et al., 2017). Population parameters were estimated using the First-Order Conditional Estimation method with Interaction (FOCE-I) (Nguyen et al., 2017). Fixing $K_{a}$ is a necessary strategy in sparse data analysis to stabilize the estimation of CL/F and V/F, assuming that absorption kinetics remain relatively constant across the study cohort.

### 3.3 Covariate model

Using a stepwise covariate selection procedure (forward inclusion ,p<0.05 ; backward elimination, p<0.01 ), age and concomitant VPA use were retained as significant covariates on CL/F. CL/F decreased with increasing age. Conversely, VPA co-administration was associated with an approximately 47.7% increase in CL/F, which corresponds to lower serum concentrations. Lithium carbonate showed an initial signal in univariate forward screening. Still, it was not retained after multivariable evaluation, indicating that its effect was not robust enough for inclusion in the final model (Table 2). The final model parameters were defined as follows: AGE represents patients' age (years, continuous variable). VPA denotes valproate comedication status (binary categorical variable): 0 = no comedication, 1 = comedication. No significant correlation was observed between continuous VPA concentrations and the final model ETAs on CL/F (Spearman r = 0.12, p = 0.519; Supplementary Figure S1A), nor were there significant differences across stratified concentration tiers (Supplementary Figure S1B), confirming that treating VPA as a binary covariate was adequate. The final model RSDs for structural parameters were <30%, confirming precise estimation.

$$\frac{CL}{F_{i}}=339\times \left[1-0.0125\times \left(\text{AGE}-22\right)\right]\times \left(1+0.477\times \text{VPA}\right)\times e^{\eta_{i}}$$

$$\frac{V}{F_{i}}=13600$$

$$K_{a}=0.679{\;h}^{-1}$$

**TABLE 2:** Processes of forward inclusion and backward exclusion.

Model number	Explanation	OFV	ΔOFV	P-value
Forward inclusion				
1	Base model	873.88	—	—
2	Model 1 +AGE	865.43	−8.44	<0.05
3	Model 1 +VPA	867.79	−6.09	<0.05
4	Model 1 +Li_2_CO_3_	869.08	−4.79	<0.05
5	Model 2 +VPA	858.45	−6.98	<0.05
6	Model 2 +Li_2_CO_3_	865.04	−0.39	>0.05
Backward elimination				
7	Model 5-AGE	867.78	+9.33	<0.01
8	Model 5-VPA	865.43	+6.98	<0.01

### 3.4 Model evaluation

**FIGURE 1:**
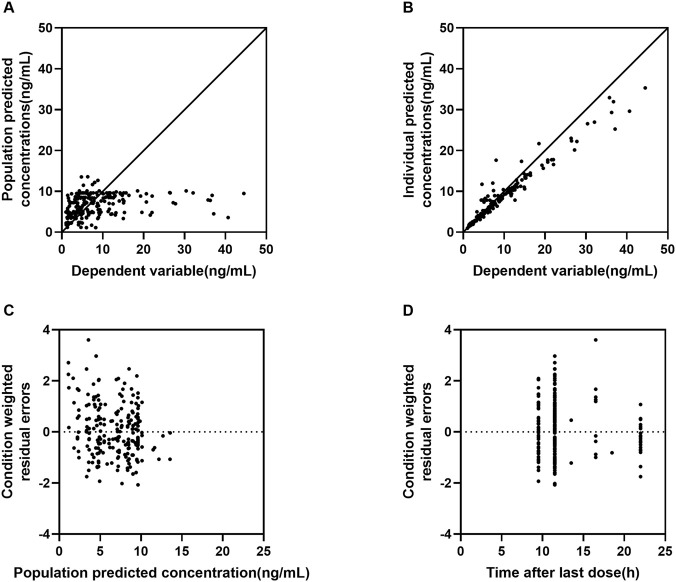
Goodness-of-fit (GOF) plots of lurasidone PPK base model. Note: **(A)** dependent variable (DV) versus population prediction (PRED); **(B)** DV versus individual prediction (IPRED); **(C)** conditional weighted residuals (CWRES) versus PRED; **(D)** CWRES versus time after last dose.

**FIGURE 2:**
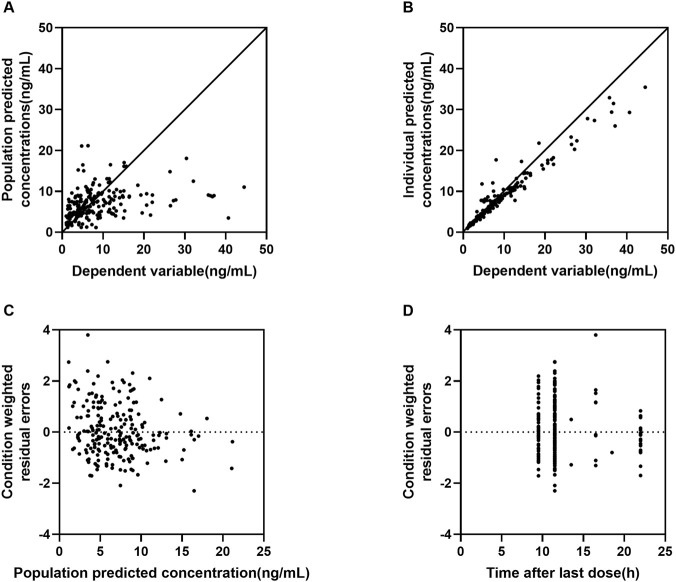
Goodness-of-fit (GOF) plots of lurasidone PPK final model. Note: **(A)** dependent variable (DV) versus population prediction (PRED); **(B)** DV versus individual prediction (IPRED); **(C)** conditional weighted residuals (CWRES) versus PRED; **(D)** CWRES versus time after last dose.

**FIGURE 3:**
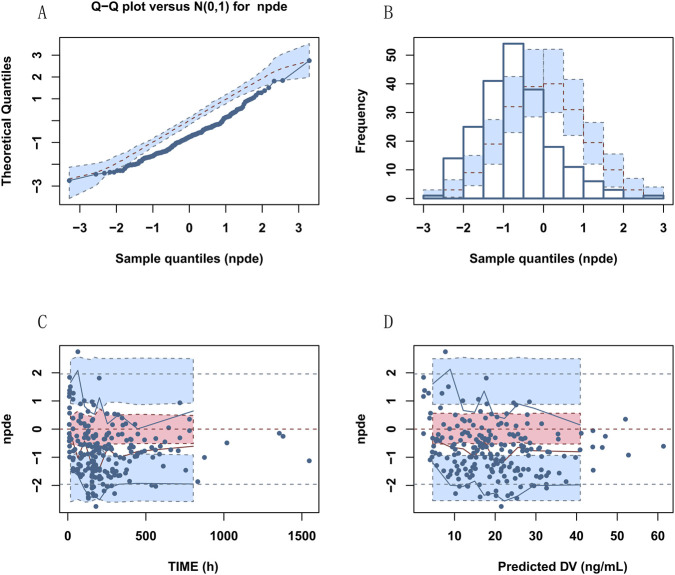
Normalized prediction distribution error (NPDE) diagnostics of lurasidone PPK final model. Note: **(A)** Q–Q plot of NPDE versus the standard normal distribution; **(B)** histogram of NPDE with the standard normal density overlay; **(C)** NPDE versus sampling time; **(D)** NPDE versus population-predicted concentration (PRED).

Goodness of fit (GOF) plots (DV vs. PRED, DV vs. IPRED, CWRES vs. PRED, CWRES vs. TIME) demonstrated no systematic bias and showed a random distribution of residuals around zero (Figures 1, 2). However, considering the 41% eta-shrinkage for V/F, the DV versus IPRED plot should be interpreted with caution, as individual estimates naturally shrink toward the population typical values due to data sparsity in the distribution phase. Additionally, specific diagnostics using individual weighted residuals (IWRES) versus time after dose (Supplementary Figure S2) clearly highlighted the scarcity of data points in the early distribution phase (0–8 h), which justifies the necessary selection of a parsimonious one-compartment structure. Normalized predictive distribution error (NPDE) diagnostics approximated a standard normal distribution, with no significant trends across time or predicted concentrations (Figure 3), supporting adequate predictive performance. Furthermore, NPDE diagnostic plots (Figure 3) confirmed model adequacy, with minor skew attributable to lower exposures in the cohort. Non-parametric bootstrap analysis (n = 1000) confirmed model robustness: bootstrap medians closely matched the final parameter estimates, and all estimates fell within the 95% bootstrap confidence intervals. Furthermore, the bootstrap-derived RSE% values for most fixed and random effect parameters were well within acceptable limits. Notably, the RSE% for the apparent volume of distribution (V/F) was 18.2%, confirming the precision and robustness of its estimate despite the observed shrinkage (Table 3). Overall, diagnostic results support the stability and predictive reliability of the final model.

**TABLE 3:** Final model parameters and bootstrap results of lurasidone PPK.

Parameter	Final model			Bootstrap method		​
	Estimated	RSD (%)	Shrinkage (%)	Median	95% CI	RSE (%)
Ka (h-1)	0.679,FIX	—	—	0.679, FIX	—	—
CL/F (L·h-1)	339	7	—	337	283–394	8.6
V/F (L)	13600	20	—	13531	8802–19807	18.2
θCL-AGE	0.0125	16	—	0.0122	0.0062–0.0172	22.5
θCL-VPA	0.477	29	—	0.478	0.109–0.880	40.5
Random effects						​
CL/F (L·h-1)	0.505	20	19	0.480	0.307–0.673	18.7
V/F (L)	1.24	19	41	1.259	0.539–1.974	26.8
Residual error						​
Additive residual	0,FIX	—	—	0, FIX	—	—
Residual error	0.0779	24	39	0.0757	0.0411–0.1220	26.6

Ka: primary absorption rate constant; CL/F: apparent clearance; V/F: apparent volume of distribution; θ_CL-AGE_: effect of age on apparent clearance; θ_CL-VPA_: effect of combined valproic acid on apparent clearance; CV: coefficient of variation.

### 3.5 Model-based simulations

#### 3.5.1 Dose–exposure and therapeutic window (15–40 ng/mL)

**FIGURE 4:**
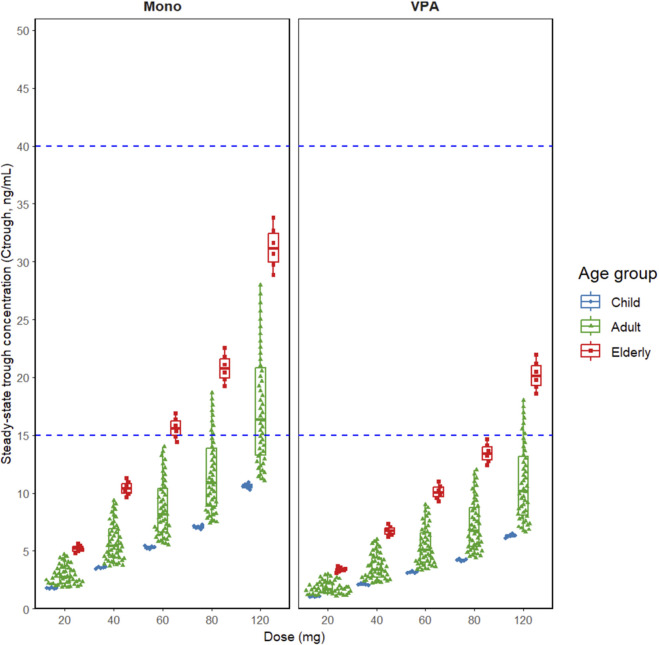
Steady-state trough concentration distributions by dose, age group. Note: (adolescent/adult/elderly) and VPA co-administration (Mono vs. VPA); dashed blue lines indicate the AGNP window lower bound (15 ng/mL) and the AGNP window upper bound (40 ng/mL).

Simulated steady-state trough concentration distributions by dose, age group, and VPA co-administration are shown in Figure 4. Simulated steady-state trough concentrations were highest in the elderly (≥65 years), followed by adults (18–64 years) and adolescents (13–17 years), inversely correlating with clearance. Based on the AGNP recommended therapeutic window (15–40 ng/mL), adolescent median concentrations and percentiles remained below 15 ng/mL at all simulated doses under monotherapy. For adults, while median values generally remained below 40 ng/mL, the upper quartiles or maxima approached or entered the 15–40 ng/mL window at mid-to-high doses (particularly 80–120 mg). The elderly group at 120 mg showed the highest exposure, with medians and upper quartiles closest to the therapeutic window. Consistent with the 47.7% increase in clearance identified in the covariate model, VPA co-administration shifted the simulated steady-state concentration distributions significantly downward, reducing the proportion of individuals reaching the 15–40 ng/mL range across all age groups; under VPA cotreatment, no upper quartiles exceeded 40 ng/mL. The preserved age ranking suggests that age-related decreases in clearance contribute to higher exposure in the elderly, while the increased inter-individual variability observed in the 80–120 mg range implies greater exposure uncertainty in this population (Figure 4).

#### 3.5.2 Exploratory internal reference range

**FIGURE 5:**
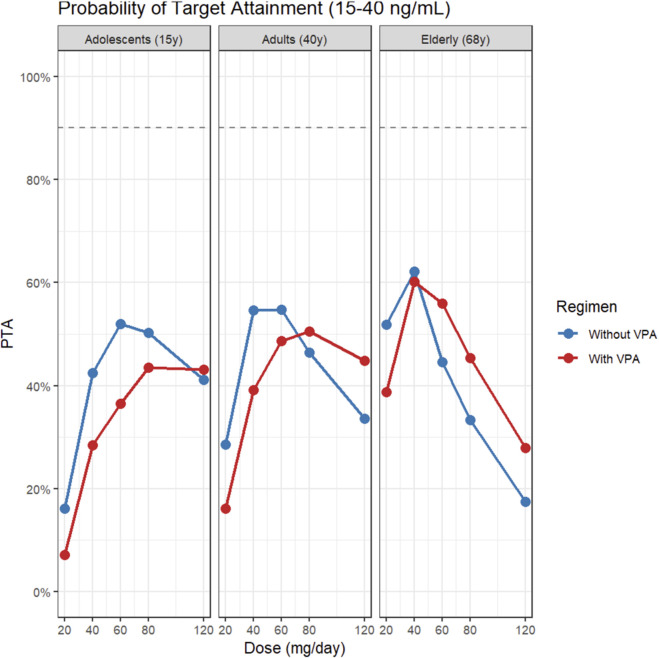
PTA for attaining the AGNP target window (15–40 ng/mL) across doses by age group and VPA co-medication. Note: adolescents (15 years), adults (40 years), and elderly (68 years) across doses; blue line = without VPA, red line = with VPA.

**FIGURE 6:**
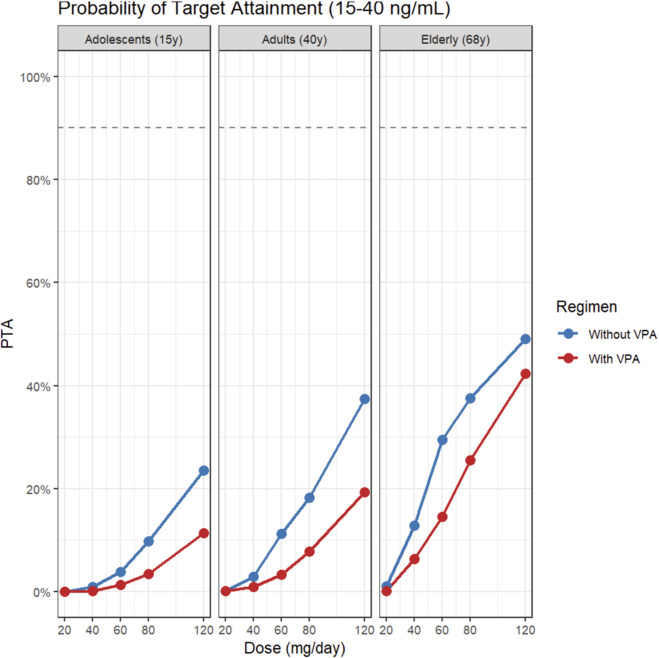
PTA for attaining the exploratory target window (3–11 ng/mL) across doses by age group and VPA co-medication. Note: adolescents (15 years), adults (40 years), and elderly (68 years) across doses; blue line = without VPA, red line = with VPA.

An exploratory internal reference range of 3–11 ng/mL was defined based on the simulated interquartile range (IQR) of the adult population in this study (Q1-Q3 ∼ 3.67–10.28 ng/mL). We subsequently calculated the probability of target attainment (PTA) for both this exploratory window and the standard AGNP window (15–40 ng/mL). Across simulated populations, PTA generally increased with dose but was consistently lower in patients co-administered VPA. Using the AGNP window (15–40 ng/mL; Figure 5), PTA was low at typical starting doses but improved with dose escalation. For a typical 40-year-old adult on monotherapy, PTA was 11% at 40 mg/day, 18% at 80 mg/day, and 37% at 120 mg/day; however, with VPA co-administration, these values dropped to 2%, 7%, and 19%, respectively. Elderly patients (68 years) achieved higher attainment rates at equivalent doses, reaching 28% at 40 mg/day and 50% at 120 mg/day under monotherapy. In contrast, using the exploratory window (3–11 ng/mL; Figure 6), PTA values were higher and typically peaked at intermediate doses. For adults on monotherapy, PTA rose from 28% at 20 mg/day to 55% at 40–80 mg/day, before declining to 33% at 120 mg/day as concentrations exceeded the upper limit. Under VPA co-administration, PTA for adults was 16% at 40 mg/day, peaking at 39% at 80 mg·day^-1^. Notably, for elderly patients on monotherapy, PTA for this lower window dropped sharply from 62% at 40 mg/day to 17% at 120 mg/day, reflecting a shift where the majority of subjects exceeded the 11 ng/mL upper limit. Overall, VPA co-administration consistently shifted the PTA curves downward across both target windows and all age groups (Figures 5, 6; Supplementary Table S4).

## 4 Discussion

### 4.1 Summary of findings

We developed a one compartment PPK model with first order absorption and elimination using FOCE I to describe lurasidone pharmacokinetics in Chinese psychiatric patients. The final model was stable and predictive, identifying age and VPA as primary covariates influencing CL/F. Typical population estimates were 13600 L and CL/F = 339 L/h. Due to the sparse sampling design, which consisted predominantly of steady-state trough concentrations, the K_a_ was fixed at 0.679 h^-1^. A subsequent sensitivity analysis confirmed that this assumption did not significantly bias the V/F estimation. Furthermore, it demonstrated that the relatively high shrinkage (41%) observed for V/F is an inherent limitation of sparse trough sampling, rather than an artifact of the fixed K_a_. In contrast, the shrinkage for CL/F was notably low (19%), ensuring the robust identification of clearance-related covariates. These clearance values are broadly consistent with recent findings by Dai et al., who reported a typical CL/F of approximately 312 L/h in a Chinese cohort, supporting the plausibility of our parameter estimates despite differences in model structure (Hu et al., 2017; Dai et al., 2026).

### 4.2 Age as a determinant and dosing implications

Age emerged as the most significant determinant of clearance in our analysis. Given that lurasidone is predominantly metabolized by hepatic CYP3A4 with minimal renal excretion, renal function markers (e.g., CrCL) were evaluated but not retained as covariates, CrCL was evaluated during covariate screening but was not retained (ΔOFV = 2.974, p > 0.05). Therefore, age in our cohort acts as a composite surrogate for hepatic metabolic capacity. The reduced clearance in the elderly reflects age-related decreases in hepatic blood flow and CYP3A4 activity, whereas the higher clearance in adolescents stems from their relatively larger liver volume and regional blood flow (Tan et al., 2015; Horvat et al., 2023). Importantly, our data showed that the elderly received slightly lower median doses (50 mg/day) than younger patients (60 mg/day), confirming that their elevated exposure is conclusively driven by these intrinsic pharmacokinetic changes (i.e., reduced clearance) rather than higher clinical dosing. Simulations demonstrated that at typical doses (20–120 mg/day), elderly patients (≥65 years) achieve substantially higher steady-state concentrations—approximately three times those of adolescents or young adults—thereby risking exposure levels that exceed the upper bounds of the therapeutic window. This finding complements the work of Dai et al., whose cohort was predominantly younger and focused on genotype effects. While Dai et al. (Dai et al., 2026) emphasized CYP3A4 polymorphisms, our study highlights that in a broader, clinically heterogeneous hospitalized population, age serves as a readily identifiable proxy for reduced clearance. Consequently, fixed dosing across age groups may be suboptimal; a “start low, go slow” strategy with closer clinical monitoring and early TDM may be particularly useful for elderly patients (Solh et al., 2025).

### 4.3 Impact of VPA co-medication

A pivotal finding of our analysis is that concomitant VPA increased CL/F of lurasidone by 47.7%, leading to significantly reduced total serum concentrations. This result appears counter-intuitive, as VPA is classically characterized as a CYP inhibitor. However, our observation aligns with reports that VPA can paradoxically lower serum concentrations of certain antipsychotics (Cerveny et al., 2007; Diaz et al., 2014). While Dai et al. (Dai et al., 2026) did not identify VPA as a significant covariate despite 14.2% usage, this discrepancy may stem from differences in sample size or covariate selection criteria. Mechanistically, we propose two plausible pathways for this interaction. First, VPA may trigger transcriptional upregulation of CYP3A4 and multidrug resistance protein 1 (MDR1) via pregnane X receptor (PXR)-mediated induction, effectively accelerating metabolism. Second, and perhaps more critical for interpretation, is protein binding displacement. Both lurasidone (>99%) and VPA (∼90%) are highly protein-bound. VPA competition may displace lurasidone from serum binding sites, transiently increasing its free fraction, which is then more rapidly cleared by the liver. Since clinical assays measure total rather than free drug concentration, this manifests as an apparent increase in clearance. This distinction has profound clinical implications. If protein binding displacement is the dominant mechanism, the concentration of free (pharmacologically active) lurasidone may remain sufficient despite the drop in total serum levels. In this scenario, blind dose escalation based solely on “sub-therapeutic” total concentrations could paradoxically increase the risk of toxicity from unbound drug (Benet and Hoener, 2002). Therefore, we strongly advocate that clinicians interpret low lurasidone levels with caution in VPA-co-medicated patients, prioritizing clinical response (e.g., PANSS reduction) over rigid adherence to reference ranges.

### 4.4 Therapeutic window and dose-response considerations

A critical finding of our study is the substantial discrepancy between the observed concentrations in Chinese patients and the AGNP recommended therapeutic window (15–40 ng/mL). In our cohort, serum concentrations at standard doses (20–120 mg/day) were predominantly distributed within the range of 3–11 ng/mL, with a median trough of approximately 6.3 ng/mL. This “low exposure” phenomenon is not isolated but aligns with emerging evidence from other Asian populations. For instance, a Taiwanese study suggested a lower reference range of 10–15 ng/mL (Huang and Lin, 2022), and recent findings in Chinese patients with bipolar depression reported effective therapeutic concentrations as low as 4–6 ng/mL (Dai et al., 2026). The stark contrast between our real-world data and the AGNP guidelines warrants critical examination of the underlying mechanisms. While genetic polymorphisms in CYP3A4 among Han Chinese may contribute to metabolic differences, the strict caloric requirement for lurasidone absorption (≥350 kcal) is likely the dominant factor. The AGNP reference range is largely derived from controlled clinical trials where high-calorie meals are mandated. In contrast, our study reflects a 'real-world' inpatient setting where appetite suppression-a common symptom of psychiatric urgency or medication side effect-often compromises meal compliance. Although medication was administered with food, the caloric content may not have consistently reached the 350 kcal threshold required for optimal bioavailability (Preskorn et al., 2013). Consequently, the low exposure observed likely reflects reduced bioavailability due to suboptimal dietary intake rather than intrinsic hyper-clearance. This suggests that the AGNP reference range, established under optimized trial conditions, may be unrealistically high for routine clinical practice. Crucially, failure to reach the 15–40 ng/mL window does not necessarily indicate therapeutic failure. Our simulations suggest that maintaining concentrations within a locally calibrated range of 3–11 ng/mL may be sufficient for clinical stability in this population, though prospective exposure-response studies are needed to validate this lower threshold.

### 4.5 Limitations

The study has several limitations. First, the relatively small sample size and sparse sampling design (primarily steady-state trough concentrations) limited the characterization of both the absorption and early distribution phases (Ka was fixed). This intrinsic data sparsity resulted in a 41% shrinkage for V/F and restricted our structural model to a one-compartment framework; nevertheless, the remarkably low shrinkage (19%) for apparent clearance (CL/F) confirmed the high reliability of individual clearance estimates for TDM purposes. Second, while patients in our cohort descriptively achieved clinical stability without documented severe extrapyramidal symptoms (EPS), the absence of direct pharmacodynamic endpoints (e.g., structured PANSS scores) means our proposed 3–11 ng/mL exploratory range and PTA analyses relate only to pharmacokinetic concentration windows rather than validated clinical outcomes. Third, as with any retrospective study utilizing routine clinical data, our analysis heavily relies on electronic medical records. This inherent reliance makes the study susceptible to documentation entry errors (e.g., inaccuracies in recorded dosing times, actual administered doses, or exact blood sampling times). Such documentation errors could contribute to increased RUV or introduce bias into the pharmacokinetic parameter estimates (Broeker et al., 2019). Additionally, the limited number of paired observations (n = 39) may lack the statistical power to detect subtle concentration-dependent effects of VPA. Furthermore, VPA co-prescription is often driven by greater disease severity—a potential confounder. Although our supervised inpatient setting minimizes non-adherence, the exact impact of underlying metabolic states or disease severity on pharmacokinetics could not be fully adjusted for. Future prospective studies are warranted to disentangle true pharmacokinetic interactions from disease-driven physiological changes. Finally, while age was successfully identified as a continuous covariate affecting CL/F, the proportion of elderly patients (≥65 years) in our current cohort was severely limited (n = 4). Consequently, while the wide continuous age span (13–70 years) provided sufficient data to capture the overall trend of age-related clearance reduction, the specific simulation results for the elderly population involve a degree of extrapolation and lack statistical power. These specific predictions should be considered hypothesis-generating and interpreted with clinical caution.

### 4.6 Clinical implications and recommendations

Our findings support stratified dosing strategies based on age and comedication status. Given the reduced clearance observed in elderly patients, lower starting doses and closer monitoring may be warranted to avoid excessive exposure. In contrast, patients receiving VPA showed a significantly increased apparent clearance (approximately 48%), suggesting reduced total exposure and potentially different dose requirements. However, whether dose escalation is necessary should be determined cautiously in light of clinical response, tolerability, and TDM results. Although the AGNP therapeutic reference range (15–40 ng/mL) is the current standard, the lower concentrations observed in our cohort (3–11 ng/mL) were consistent with clinical stability in our practice and in external reports (Preskorn et al., 2013; Yang et al., 2024; Dai et al., 2026; Huang and Lin, 2022), this suggests that strict adherence to the AGNP lower limit may lead to unnecessary dose escalation in Chinese patients. This perspective is consistent with the concept of target concentration intervention, which emphasizes individualized concentration targets linked to clinical response rather than rigid adherence to a universal therapeutic range (Holford, 2001; Schoretsanitis et al., 2020).

## 5 Conclusion

Lurasidone exhibited high inter-individual variability in Chinese psychiatric inpatients, with steady-state concentrations generally falling below the AGNP reference range (15–40 ng/mL). Concomitant VPA significantly increased apparent clearance (47.7%), likely driven by a combination of enzyme induction and protein binding displacement. Consequently, clinicians should interpret “low” total lurasidone concentrations with caution in patients receiving VPA, differentiating between reduced systemic exposure and assay artifacts caused by protein displacement, to avoid unnecessary dose escalation.

## Data Availability

The original contributions presented in the study are included in the article/Supplementary Material, further inquiries can be directed to the corresponding authors.
