# Supplementary figures and images for: Impact of valproate co-medication and age on lurasidone exposure: a population pharmacokinetic study and real-world evaluation in Chinese psychiatric inpatients
Source: Front Pharmacol. 2026 May 12;17:1810528. doi: 10.3389/fphar.2026.1810528 (PMC13201226; doi:10.3389/fphar.2026.1810528)

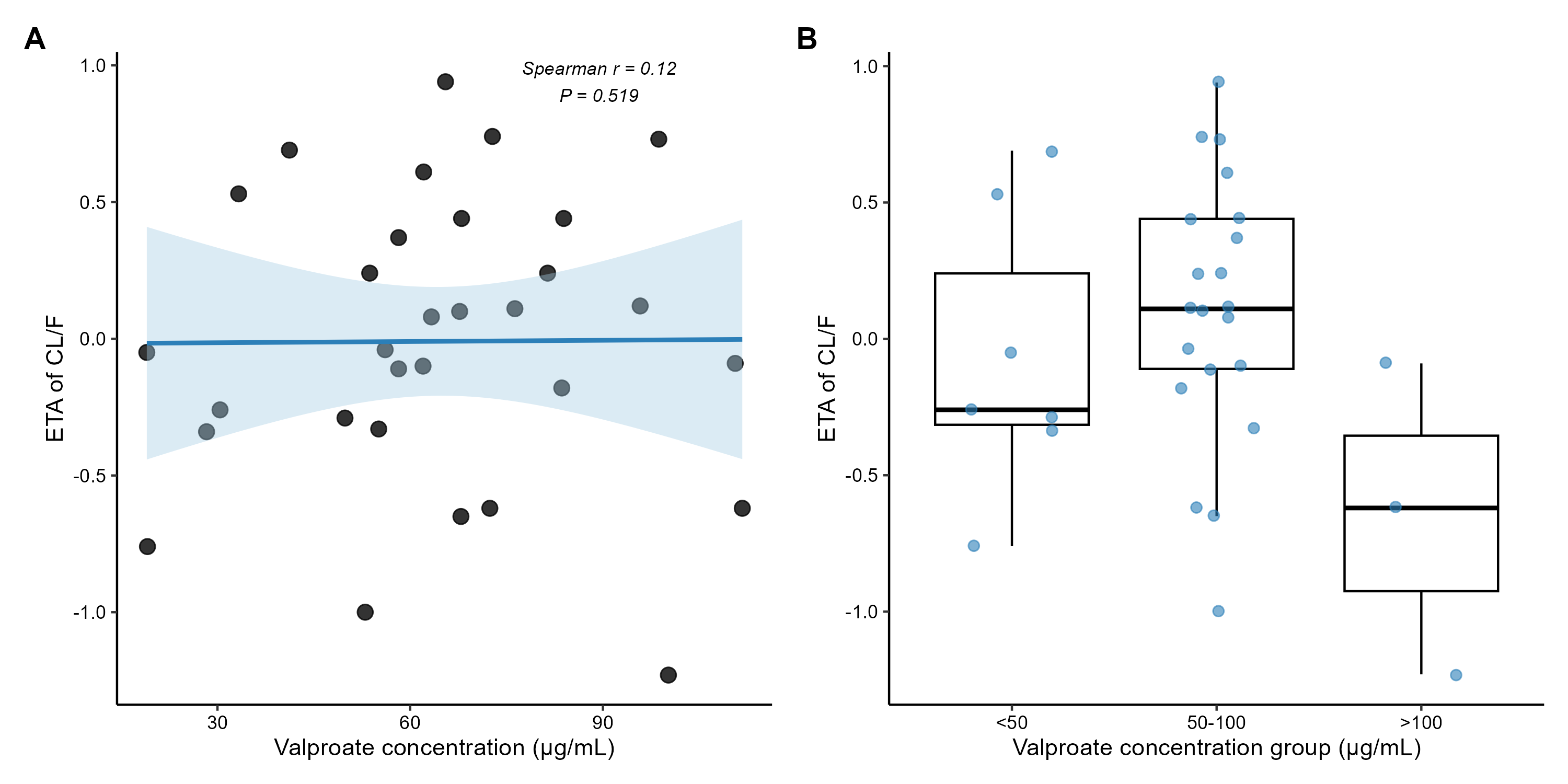

Supplement: Supplementary file 1 [file Image1.TIFF]

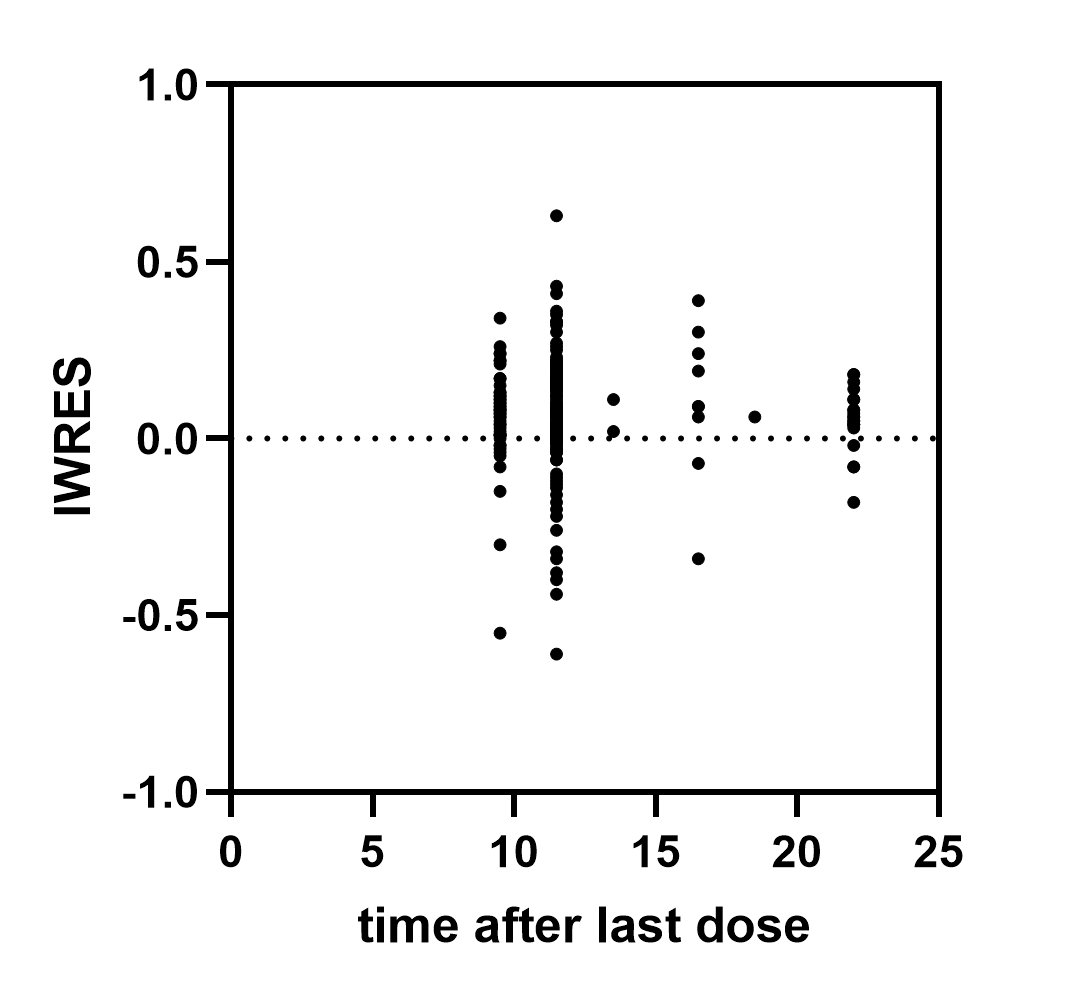

Supplement: Supplementary file 3 [file Image2.TIF]
