# Supplementary material for: Impact of valproate co-medication and age on lurasidone exposure: a population pharmacokinetic study and real-world evaluation in Chinese psychiatric inpatients
Source: Front Pharmacol. 2026 May 12;17:1810528. doi: 10.3389/fphar.2026.1810528 (PMC13201226; doi:10.3389/fphar.2026.1810528)
Supplement: Supplementary file 2 [file Table1.docx]

**Supplementary Table S1 Results of the Ka Sensitivity Analysis based on the Final Model**

| Parameters | Ka - 20%  (0.543 h⁻¹) | Original Ka  (0.679 h⁻¹) | Ka + 20%  (0.815 h⁻¹) |
| --- | --- | --- | --- |
| Objective Function Value (OFV) | 856.562 | 858.451 | 859.712 |
| CL/F (L/h) | 343 | 339 | 336 |
| V/F (L) | 13800 | 13600 | 13700 |
| Shrinkage of V/F (%) | 41% | 41% | 41% |
| CL-AGE effect | 0.0126 | 0.0125 | 0.0125 |
| CL-VPA effect | 0.479 | 0.477 | 0.474 |
