# Supplementary material for: Impact of valproate co-medication and age on lurasidone exposure: a population pharmacokinetic study and real-world evaluation in Chinese psychiatric inpatients
Source: Front Pharmacol. 2026 May 12;17:1810528. doi: 10.3389/fphar.2026.1810528 (PMC13201226; doi:10.3389/fphar.2026.1810528)
Supplement: Supplementary file 4 [file Table2.docx]

**Supplementary Table S2 Number of patients and lurasidone concentrations by age category**

| Age Category | Age Range (years) | Number of Patients, n (%) | Number  of Concentrations,  n (%) |
| --- | --- | --- | --- |
| Adolescents | 13-17 | 60 (38.5%) | 68 (32.1%) |
| Adults | 18-64 | 92 (59.0%) | 139 (65.6%) |
| Elderly | ≥65 | 4 (2.6%) | 5 (2.4%) |
| Total | 13-70 | 156 (100%) | 212 (100%) |
