# Supplementary material for: Impact of valproate co-medication and age on lurasidone exposure: a population pharmacokinetic study and real-world evaluation in Chinese psychiatric inpatients
Source: Front Pharmacol. 2026 May 12;17:1810528. doi: 10.3389/fphar.2026.1810528 (PMC13201226; doi:10.3389/fphar.2026.1810528)
Supplement: Supplementary file 5 [file Table3.docx]

**Supplementary Table S3 Dosage statistics by age group**

| Age Group | N | Median Dose (mg/day) | Dose Range (mg/day) | Mean ± SD (mg/day) |
| --- | --- | --- | --- | --- |
| Overall | 156 | 60 | 20-120 | 57.69±20.69 |
| Adolescents (13-17 years) | 60 | 60 | 20-80 | 54.67±20.12 |
| Adults (18-64 years) | 92 | 60 | 20-120 | 60.00±20.75 |
| Elderly (≥65 years) | 4 | 50 | 20-80 | 50.00±25.82 |
