# Supplementary material for: Impact of valproate co-medication and age on lurasidone exposure: a population pharmacokinetic study and real-world evaluation in Chinese psychiatric inpatients
Source: Front Pharmacol. 2026 May 12;17:1810528. doi: 10.3389/fphar.2026.1810528 (PMC13201226; doi:10.3389/fphar.2026.1810528)
Supplement: Supplementary file 6 [file Table4.docx]

**Supplementary Table S4 Effects of age group, VPA co-administration, and dose on PTA (15-40 ng/mL and 3-11 ng/mL)**

| Age group | VPA status | Dose (mg/day) | PTA  (15-40 ng/mL) | PTA  (3–11 ng/mL) |
| --- | --- | --- | --- | --- |
| Adolescents (15 y) | Without VPA | 40 | 9% | 42% |
| Adolescents (15 y) | Without VPA | 80 | 23% | 51% |
| Adolescents (15 y) | Without VPA | 120 | 48% | 41% |
| Adolescents (15 y) | With VPA | 40 | 1% | 28% |
| Adolescents (15 y) | With VPA | 80 | 10% | 36% |
| Adolescents (15 y) | With VPA | 120 | 43% | 44% |
| Adults (40 y) | Without VPA | 20 | 0% | 28% |
| Adults (40 y) | Without VPA | 40 | 11% | 55% |
| Adults (40 y) | Without VPA | 80 | 18% | 55% |
| Adults (40 y) | Without VPA | 120 | 37% | 33% |
| Adults (40 y) | With VPA | 40 | 2% | 16% |
| Adults (40 y) | With VPA | 80 | 7% | 39% |
| Adults (40 y) | With VPA | 120 | 19% | 28% |
| Elderly (68 y) | Without VPA | 20 | 0% | 52% |
| Elderly (68 y) | Without VPA | 40 | 28% | 62% |
| Elderly (68 y) | Without VPA | 80 | 36% | 45% |
| Elderly (68 y) | Without VPA | 120 | 50% | 17% |
| Elderly (68 y) | With VPA | 40 | 6% | 38% |
| Elderly (68 y) | With VPA | 80 | 25% | 46% |
| Elderly (68 y) | With VPA | 120 | 42% | 27% |
